# Molecular evolution analysis of *MYB5* in Brassicaceae with specific focus on seed coat color of *Brassica napus*

**DOI:** 10.1186/s12870-023-04718-6

**Published:** 2024-01-16

**Authors:** Guoqiang Dai, Yi Liu, Wenjie Shen, Bo Zhu, Lunlin Chen, Daozong Chen, Chen Tan

**Affiliations:** 1 College of Life Sciences, Ganzhou Key Laboratory of Greenhouse Vegetable, Gannan Normal University, Ganzhou, 341000 China; 2 Nanchang Branch of National Center of Oilcrops Improvement, Jiangxi Province Key Laboratory of Oil Crops Biology, Crops Research Institute of Jiangxi Academy of Agricultural Sciences, Nanchang, 330200 China

**Keywords:** Brassicaceae, *MYB5*, Homologous gene, Seed coat, *Brassica napus*

## Abstract

**Background:**

MYB transcription factors are splay a vital role in plant biology, with previous research highlighting the significant impact of the R2R3-MYB-like transcription factor *MYB5* on seed mucilage biosynthesis, trichome branching, and seed coat development. However, there is a dearth of studies investigating its role in the regulation of proanthocyanidin (PA) biosynthesis.

**Results:**

In this study, a total of 51 *MYB5* homologous genes were identified across 31 species belonging to the Brassicaceae family, with particular emphasis on *Brassica napus* for subsequent investigation. Through phylogenetic analysis, these genes were categorized into four distinct subclasses. Protein sequence similarity and identity analysis demonstrated a high degree of conservation of *MYB5* among species within the Brassicaceae family. Additionally, the examination of selection pressure revealed that *MYB5* predominantly underwent purifying selection during its evolutionary history, as indicated by the Ka/Ks values of all *MYB5* homologous gene pairs being less than one. Notably, we observed a higher rate of non-synonymous mutations in orthologous genes compared to paralogous genes, and the Ka/Ks value displayed a stronger correlation with Ka. In *B. napus*, an examination of expression patterns in five tissues revealed that *MYB5* exhibited particularly high expression in the black seed coat. The findings from the WGCNA demonstrated a robust correlation between *MYB5* and *BAN*(*ANR*) associated with PA biosynthesis in the black seed coat, providing further evidence of their close association and co-expression. Furthermore, the results obtained from of the analysis of protein interaction networks offer supplementary support for the proposition that *MYB5* possesses the capability to interact with transcriptional regulatory proteins, specifically *TT8* and *TT2*, alongside catalytic enzymes implicated in the synthesis of PAs, thereby making a contribution to the biosynthesis of PAs. These findings imply a plausible and significant correlation between the nuique expression pattern of *MYB5* and the pigmentation of rapeseed coats. Nevertheless, additional research endeavors are imperative to authenticate and substantiate these findings.

**Conclusions:**

This study offers valuable insights into the genetic evolution of Brassicaceae plants, thereby serving as a significant reference for the genetic enhancement of Brassicaceae seed coat color.

**Supplementary Information:**

The online version contains supplementary material available at 10.1186/s12870-023-04718-6.

## Background

The MYB transcription factor family in plants is known for its large size and is characterized by the presence of 1-4 R repeat sequences, with each repeat sequence consisting of approximately 52 amino acids. Based on the number of R repeats, MYB transcription factors can be classified into four types: 1R-MYB, R2R3-MYB, 3R-MYB and 4R-MYB [1]. The discovery of the first MYB transcription factor, *COLORED1* (*C1*), which regulates anthocyanin synthesis in the maize (*Zea mays*) grain aleurone layer dates back more than 30 years [2]. Since then, extensive research has been conducted on the involvement of MYB transcription factors in various plant species, including *Arabidopsis thaliana* [3], rice (*Oryza sativa*) [4], grape (*Vitis vinifera*) [5], apple (*Malus domestica*) [6], and petunia (*Petunia hybrida*) [7], utilizing sequencing, genetic, and molecular analysis techniques. Consequently, novel insights into the regulatory mechanisms governing MYB protein activity have emerged, along with the identification of the gene expression profiles and certain target genes. Among the four types discussed, the R2R3-MYB transcription factor emerges as the most common prevalent, characterized by the presence of two consecutive R repeats [8]. Extensive research has demonstrated that the R2R3-MYB transcription factor primarily exerts its influence on the structural genes involved in the plant anthocyanin biosynthesis pathway. Additionally, it has been observed to form a transcriptional regulatory complex with bHLH transcription factors and WD40 proteins, collectively known as the MBW complex. This complex plays a crucial role in modulating the synthesis and accumulation of anthocyanin [8, 9].

Throughout the extensive process of evolution, R2R3-MYB transcription factors have displayed functional diversity and can be found in all land plants. Throughout the extensive process of evolution, the functions of R2R3-MYB transcription factor have undergone divergence, leading to the characterization of numerous R2R3-MYB proteins through genetic methods. These proteins have been found to play a role in regulating plant-specific processes, such as responses to biotic and abiotic stresses [10, 11], cell fate and identity, primary and secondary metabolism [8, 12–16] and developmental processes [17]. The R2R3-MYB transcription factor, known as MYB5, has been extensively studied and identified in various plant species. Its primary functions include the regulation of tannin biosynthesis [16], trichome elongation [12, 13], outer seed coat differentiation [13, 16] and procyanidin synthesis [18]. In *A. thaliana*, MYB5 typically forms a complex with *TT8* and *TTG1*, collectively referred to as the MBW complex, to directly target *DFR*, *ANS*, or *BAN* genes and regulate flavonoid biosynthesis in seeds [18].

The Brassicaceae family encompasses a total of 351 genera and approximately 4,000 species, establishing it as a prominent constituent within the plant kingdom [19]. Including the model plant *A. thaliana*, oil crops *Brassica napus*, *B. juncea*, vegetable crops *B. rapa*, *B. oleracea*, *Raphanus sativus*, medicinal plants *Isatis indigotica* and *Lepidium apetalum*, which has important research and utilization value. Of these, *B. napus* is the third oil crop cultivated globally, contributes approximately 15% to the overall production of vegetable oil, thereby playing a significant role in the provision of edible oil, biofuel, and animal feed [20]. Enhancing oil content, refining oil quality, and developing high-yielding rapeseed varieties have been the primary objectives of breeding efforts in recent decades [21–24]. Previously, breeders engaged in cultivar selection observed that rapeseed with yellow seeds exhibited superior quality attributes, including higher oil and protein content, as well as lower levels of antinutrients such as lignin, fiber, pigments, and polyphenols [25]. Similarly, investigations conducted on *B. napus* revealed that silencing *BnTT10* gene expression resulted in elevated soluble PAs within the seeds, decreased extractable lignin, and delayed pigmentation of the seed coat [26]. The implementation of RNA interference to silence *BnTT1* family genes in rapeseed resulted in various phenotypic changes, including yellowing of the plant, thinning of the seed coat, reduction in seed weight, and alterations in the fatty acid composition of the seeds [27]. Additionally, targeted knockout of the *BnTT2* homologs in *B. napus* led to a decrease in flavonoids and an improvement in the fatty acid composition of the seeds [28]. Similarly, targeted mutation of the *BnTT8* homologs hindered the expression of phenylpropanoid and flavonoid biosynthesis genes, consequently inhibiting the accumulation of proanthocyanidins in the seed coats [29]. In our previous investigation, it was observed that *MYB5* and *TT2* exhibited differential expression patterns throughout the 8 stages of seed coat development in 6 distinct *Brassica* species. This finding implies that these genes may play a pivotal role in the modulation of seed coat color alteration [30]. Nonetheless, the precise mechanism by which *MYB5* specifically participates in the regulation of seed coat color in *B. napus* remains unreported and necessitates further investigation.

In this study, we employed the published reference genome information of 31 species belonging to the Brassicaceae family to identify homologous genes of *MYB5*. This was achieved through the utilization of homologous sequence alignment and conserved domain analysis techniques. Additionally, we conducted an analysis of the molecular evolution characteristics of *MYB5* homologous genes in Brassicaceae, which enabled us to construct an evolutionary map. Subsequently, we investigated the transcriptional regulation mechanism of the *MYB5* gene in *B. napus*, focusing on its role in seed coat color formation. Our analysis involved examining tissue-specific expression patterns and co-expression patterns. The objective of this research was to enhance our comprehension of the gene function of *MYB5*. Furthermore, this study aims to establish a basis for future investigations into the molecular mechanism by which *MYB5* specifically regulates seed coat color formation, and to offer insights for the improving the quality of rapeseed oil.

## Methods

### Retrieval and identification of *MYB5* in Brassicaceae

Here, all the protein sequences and CDS sequences of 31 Brassicaceae species were obtained from the TBGR database (http://www.tbgr.org.cn/), respectively [31]. The *MYB5* gene (*AT3G13540*) from *A. thaliana* was selected as the reference for identifying homologous genes in the Brassicaceae family. To ensure accurate identification of *MYB5* homologous genes in the 31 Brassicaceae species, the following steps were primarily employed. Firstly, initial searches were conducted using local BLASTP and local BLASTN algorithms, with a significance threshold of E < 1e - 20. Secondly, the screening process involved identifying candidate genes that exhibited a consistency rate exceeding 65% and a coverage rate surpassing 60%. Thirdly, hmmsearch [32] was employed to search for potential family genes containing the MYB domain in 31 species. This search utilized the Hidden Markov model PF00249 (HMM) of the MYB domain, which was obtained from the Pfam database (http://pfam-legacy.xfam.org/) [33]. Subsequently, the outcomes of BLAST and hmmsearch were combined, and the CDD database (https://www.ncbi.nlm.nih.gov/cdd/) was employed to forecast the conserved domain, thereby eliminating genes lacking the PLN03212 superfamily. The identified *MYB5* homologous genes mentioned earlier were utilized for subsequent phylogenetic analysis. For a comprehensive understanding of the identification procedures, refer to Pucker [34].

### Construction of a phylogenetic tree

In order to validate the findings of the IQ-TREE2 phylogenetic analysis, we utilized the *MYB5* (*MD07G0126400*) orthologous gene of apple (*Malus_domestica*, golden, ASM211411v1, http://plants.ensembl.org/index.html) as the outgroup. The protein sequences of the identified 51 *MYB5* homologous genes were subjected to comparison using MAFFT (v7.520) [35] software with default parameters. For the construction of the phylogenetic tree, we employed IQ-TREE2 software (v1.6.12) [36], with the -m parameter selecting MFP to automatically determined the optimal model and generate the tree. To validate the findings of the IQ-TREE2 phylogenetic tree, we employed the RAxML-NG software (v1.1) [37] for constructing a phylogenetic tree. The LG model was chosen for tree construction, with the --bs-trees parameter set to 1000 and the -all parameter selected to facilitate multiple search process aimed at identifying the optimal tree topology and model parameters. Subsequently, the phylogenetic tree was visualized using the iTOL online website (v6.8) [38] accessible at https://itol.embl.de/.

### *Cis*-acting elements and conservative domain analysis

All 51 identified *MYB5* homologues genes upstream of the start codon 2Kb genome sequences were extracted and used for the prediction of *cis*-acting elements in the promoter region. PlantCARE [39] website (http://bioinformatics.psb.ugent.be/webtools/plantcare/html/) was used to predict the *cis*-acting elements of *MYB5* homologues genes promoters. Then, all predicted *cis*-acting elements were classified and analyzed. The identified 51 *MYB5* homologous protein sequences were extracted and submitted to CDD online website (https://www.ncbi.nlm.nih.gov/cdd/) to obtain conserved domain information, and the parameters were default parameters. Phylogenetic trees, motifs and *cis*-acting elements were mapped by TBtools software (v1.133) [40] using phylogenetic files (from FastTree2), conserved motifs (from CDD), and cis-acting elements prediction files (from PlantCARE).

### Selective pressure analysis

The CDS sequences of all identified genes were compared using the BLAST software, with a threshold value of E < 1e-20, to identify tandem repeat pairs of *MYB5* homologous genes. Subsequently, the KaKs_Calculator software (v2.0), as described by Wang et al. [41], was utilized to estimate the synonymous substitution rate (Ks) and non-synonymous substitution rate (Ka). Duplicated genes were excluded if the Ka value is close to 0 and the Ks value exceeded 3 or was below 0.01. This exclusion was necessary due to the potential saturation risk associated with high Ks values and the possibility of unknown outcomes resulting from low sequence divergence. The Ka/Ks < 1, > 1, and = 1 indicated purifying selection, forward selection, and neutral selection, respectively [42]. The visualization and plotting of the pressure analysis data were performed using the R package. For a more comprehensive understanding of the identification methods employed, please refer to Cao et al [43].

### *MYB5 *expression analysis in *B. napus*

In order to further investigate the expression patterns of *MYB5* homologous genes in various tissues of *B. napus*, transcriptome data from *B. napus* leaves (green, purple), stems (green, purple), flower petals (orange-red, yellow), siliques (green, purple), and seed coat (yellow, black) were collected and analyzed. All RNA-seq data were obtained from the NCBI (additional Center for Biotechnology Information (NCBI) at https://www.ncbi.nlm.nih.gov/ through the following biological projects PRJNA554517 for leaves, PRJAN855492 for stems and flower petals, PRJNA734925 for siliques, and PRJNA597958 for seeds. The raw data obtained from the NCBI was initially converted into fastq format using the SRA toolkit, following which it was processed with default parameters using trimmatic (v0.39) [44]. Subsequently, the RNA-seq clean reads were aligned to the reference genome of *B. napus* (Darmor-v10, https://yanglab.hzau.edu.cn/BnIR/download?module=genomics) using the HISAT2 software (v2.1.0) [45]. The obtained millions of fragments per thousand bases (FPKM) values were then calculated using StringTie (v2.1.1) [45]. The expression histogram was generated using Excel, while the heat map was generated using TBtools software (v1.133) [40].

### Construction of co-expression network modules using association analysis modules and phenotypes

To explore the metabolite fluxes of the PA biosynthetic pathway regulated by MYB5, we employed the weighted gene co-expression network analysis (WGCNA) to establish a gene co-expression network for the transcriptional regulation of PA in various tissues of *B. napus*. The construction of co-expression networks was carried out using the WGCNA software package, specifically version R-4.3.1. Subsequently, correlation analysis was performed to determine the correlation between each co-expression module and the collected tissue data of *B. napus*, enabling the identification of differentially expressed genes (DEGs) associated with PA. These DEGs were then utilized to construct a co-expression network module. For the detailed method, refer to the articles of Cheng et al. [40]. STRING software (https://version-11-5.string-db.org/) was used to reveal a protein-protein interaction network of ‘turquoise’ module genes.

## Results

### Identification and phylogenetic analysis of *MYB5* homologous genes in Brassicaceae

To comprehensively and accurately ascertain the *MYB5* homologous genes in Brassicaceae species, the *AT3G13540* sequence from *A. thaliana* was obtained and employed as a reference to explore potential candidate genes cross the 31 Brassicaceae species with available genomic data. A total of 51 *MYB5*-related genes were discovered in these 31 Brassicaceae species, with the number of *MYB5* homologous gene copies varying from one to five (Table 1, Supplementary Table S1). A total of 21 species of Brassicaceae plants were identified, with only one homologous gene being found. Among these species, allotetraploid *B. juncea* exhibited the highest number of homologous genes, totaling five, whereas the diploid crop *B. oleracea* also possessed four homologous genes (Table 1).

**Table 1:** Genome version information and *MYB5* homologous genes in 31 Brassicaceae species

Species	Version	Blast+Hmmsearch	CDD	Result
*Aethionema_arabicum*	A.arabicum-v1.0	1	1	1
*Arabidopsis_halleri*	A.halleri-v2.2	1	1	1
*Arabidopsis_lyrata*	Lyrate -v2.1	1	1	1
*Arabidopsis_thaliana*	Thale -Araport11	1	1	1
*Arabis_alpina*	Gray -v4.0	2	1	1
*Barbarea_vulgaris*	Bittercress-v1.0	1	1	1
*Brassica juncea*	SCHZ	5	5	5
*Boechera_retrofracta*	Holboell-v1.0	1	1	1
*Boechera_stricta*	Drummond-v1.2	1	1	1
*Brassica_carinata*	Zd-1-v1.0	4	4	4
*Brassica_napus*	Darmor-v10.0	4	4	4
*Brassica_nigra*	Ni100_LR-v2.0	3	3	3
*Brassica_oleracea*	HDEM-v0.0	4	2	2
*Brassica_rapa*	Chiifu-v3.5	1	1	1
*Camelina_sativa*	Camelina-v2.0	3	3	3
*Capsella_grandiflora*	C.grandiflora-v1.0	2	1	1
*Capsella_rubella*	Red_shepherd -v1.1	1	1	1
*Cardamine_hirsuta*	Hairy -v1.0	1	1	1
*Eutrema_salsugineum*	173_v1.0	1	1	1
*Isatis_indigotica*	Woad-v1.0	1	1	1
*Leavenworthia_alabamica*	Alabama -v1.0	1	1	1
*Lepidium_meyenii*	Peruvian -v1.0	3	3	3
*Matthiola_incana*	M.incana-v1.0	1	1	1
*Microthlaspi_erraticum*	M.erraticum-v1.0	2	2	2
*Orychophragmus_violaceus*	O.violaceus-v1.0	2	2	2
*Raphanus_sativus*	Radish-v1.0	1	1	1
*Schrenkiella_parvula*	Saltwater -v1.0	1	1	1
*Sinapis_alba*	S.alba-v1.0	1	1	1
*Sinapis_arvensis*	S.arvensis-v1.0	2	2	2
*Sisymbrium_irio*	London -v1.0	1	1	1
*Thlaspi_arvense*	Field -v1.1	1	1	1
Total	31	55	51	51

**Fig. 1:**
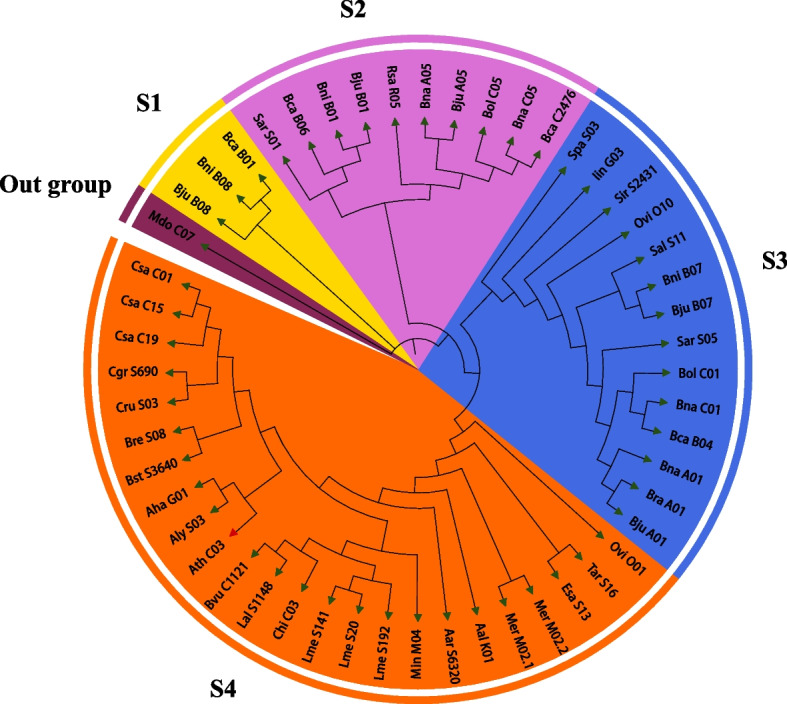
Phylogenetic relationships of MYB5 proteins between 31 Brassicaceae species. All 4 subfamilies of MYB5 proteins were well separated in different clades and represented by different colors. IQ-TREE software (v1.6.12) to build a phylogenetic tree, where the -m parameter selects MFP, automatically detects the best model and builds a tree

To study the evolutionary connections among *MYB5* homologous genes, we assigned new names to the *MYB5* homologous genes from 31 species (Supplementary Table S2) and proceeded to construct a maximum likelihood phylogenetic tree based on their protein sequences. The 51 *MYB5* homologous genes were categorized into four distinct subcategories (S1- S4) and visually represented using different colors (Fig. 1). Notably, the branches identified in *A. thaliana* were marked with green color on the evolutionary tree, indicating their placement within the S4 subcategory (Fig. 1). The S4 subcategory exhibits the highest number of *MYB5* homologous genes, totaling 24, followed by the S3 subclass which contains 14 *MYB5* homologous genes. Interestingly, within the S8 subgroup, 8 out of the 14 *MYB5* orthologs were identified in *Brassica* species, while 8 out of the 10 *MYB5* orthologs within the S2 subgroup were also found in *Brassica* species. This observation suggests that the *MYB5* gene in *Brassica* species underwent duplication and diverged at an early stage during an early developmental phase. Furthermore, the *MYB5* genes derived from *R. sativus*, *S. arvensis*, *S. parvula*, *S. alba*, *S. irio*, *O. violaceus*, and *I. indigotica* exhibit a closer clustering with the *MYB5* genes of *Brassica* crops, aligning with the evolutionary analysis findings of Brassicaceae species as reported by Yang et al. [46]. In general, the evolutionary relationship observed among the *MYB5* homologous genes across the 31 species concurs with the phylogenetic outcomes reported for Brassicaceae by Nikolov et al. [47].

### Conserved motifs and cis-acting element analysis of *MYB5* homologous genes

**Fig. 2:**
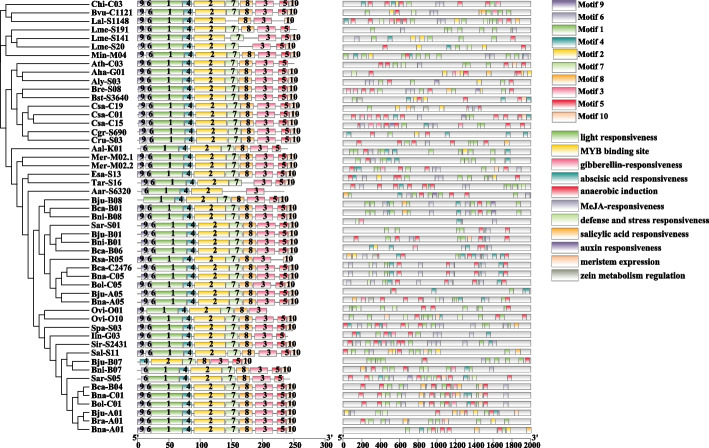
Phylogenetic, conserved motifs and promoter characteristics analysis of MYB5. Promoter sequences (-2,000 bp) of 51 *MYB5* homologous genes were analyzed using PlantCARE. Different shapes and colors represent different elements, PAFM was used for conserved domain prediction, and phylogenetic, promoter characteristics, gene structure, conserved motifs have been drawn by TBtools

To identify the functional differences of *MYB5* homologous genes in 31 Brassicaceae species, we analyzed the *cis*-acting elements in the promoters of 51 *MYB5* homologous genes with PlantCARE software. There are 11 kinds of *cis*-acting elements were found in the promoter region of the *MYB5* homologous genes, and most of them are involved in plant growth and metabolism, environmental and hormonal signal responses response, and MYB binding site (Fig. 2, Supplementary Table S3). Among the 11 types of cis-acting elements, light responsiveness, auxin responsiveness, gibberellin-responsiveness, abscisic acid responsiveness, MeJA-responsiveness, defense and stress responsiveness, salicylic acid responsiveness, and auxin responsiveness are involved in environmental and hormonal signal responses, indicating that the *MYB5* homologous genes could be induced by environmental and hormonal to regulate plant growth and development. Anaerobic induction, abscisic acid responsiveness, defense and stress responsiveness are the main members of the regulation of biological and abiotic stress. At the same time, a *cis*-element related to meristem expression was found in all 51 *MYB5* homologous genes promoter, indicating that *MYB5* may be involved in the regulation of plant growth and development by regulating the expression of meristem genes. In addition, we also found that the MYB binding site is distributed in the promoters of most *MYB5* homologous genes, indicating that MYB5 may combine with other MYB transcription factors to participate in the regulation of metabolic pathways. Our results show that during the duplication of *MYB5* gene, the *cis*-element in the promoter region between homologous genes may be mutated, resulting in the change of *cis*-acting element, and the change of *cis*-element may lead to different expression levels of homologous genes, resulting in differentiation of gene function. However, these speculations need to be further verified by follow-up experiments.

### Positive selection in *MYB5* homologous genes

**Fig. 3:**
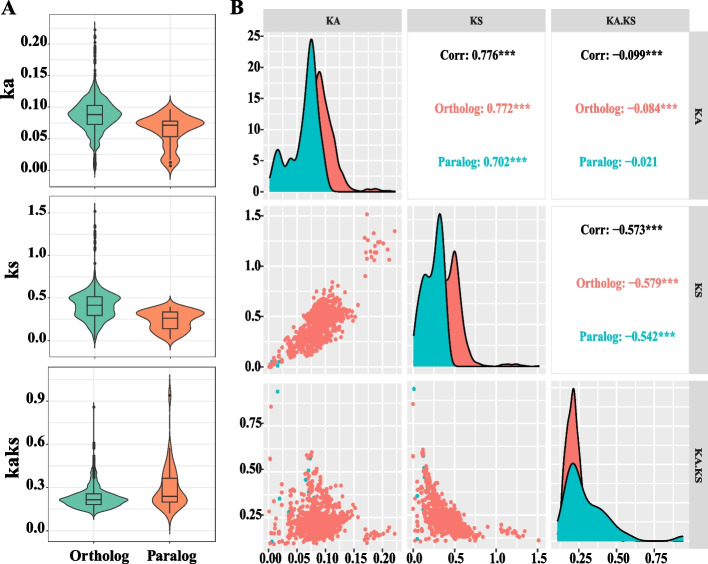
Comparison of substitution rates between orthologous and paralogous MYB5 genes among 31 Brassicaceae species. **A** Comparative analysis of Ka, Ks and Ka/Ks of paralogous and orthologous MYB5 genes. **B** Correlation analysis between Ka, Ks and Ka/Ks of paralogous and orthologous MYB5 genes

To explore the impact of selection pressure on the evolutionary process of *MYB5* homologous genes, the Ks and Ka values of ortholog/paralog pairs within the Brassicaceae family were computed (Fig. 3, Supplementary Table S4). The analysis revealed that out of the total 2467 gene pairs, all exhibited Ka/Ks ratios below 1, with 30 gene pairs falling within the range of 0.5 to 0.99, and the remaining 2437 gene pairs displaying Ka/Ks ratios < 0.5. Notably, all 2467 gene pairs had Ka values < 1, with the highest value recorded as 0.22 (Supplementary Table S5), indicating a remarkably low occurrence of non-synonymous mutations in the *MYB5* homologous genes throughout their evolutionary history. Concurrently, within the set of 2467 gene pairs, 35 pairs (1.42%) exhibited a Ks value > 1, while 705 pairs (28.58 %) displayed a Ks value ranging from 0.5 to 0.99 (Supplementary Table S4). These findings suggest a substantial occurrence of synonymous mutations in *MYB5* homologous genes, indicating their remarkable conservation and the influence of robust purifying selection throughout the course of evolution.

Subsequently, an analysis was conducted on the selection pressure exerted on *MYB5* orthologs and paralogs genes (Fig. 3). The results revealed that the average values of *MYB5* orthologous genes were 0.08753, 0.41296, and 0.2308, whereas the average Ka, Ks, and Ka/Ks values of paralogous genes were 0.06259, 0.23772, and 0.30224 (Fig. 3A). These findings suggest that the synonymous mutation rate and non-synonymous mutation rate of orthologous genes are higher than those of paralogous genes, indicating a greater accumulation of variation during the evolutionary process. However, it is observed that the Ka/Ks value of paralogous genes surpasses that of orthologous genes, suggesting that paralogous genes have experienced more pronounced selection pressure and are subject to restricted rates of evolution. Subsequently, an examination of the correlation between Ks, Ka, and Ka/Ks values of orthologous and paralogous genes of *MYB5* was conducted (Fig. 3B). The findings indicate a positive correlation between the Ka value and Ks value in both orthologous and paralogous genes. Conversely, a negative correlation is observed between the Ka value and Ks value with respect to the Ka/Ks values of both orthologous and paralogous genes. Furthermore, the strength of this correlation is primarily influenced by the Ks value. Overall, these results provide additional evidence supporting the notion that purifying selection primarily drives interspecies evolutionary pressure in *MYB5* homologous genes, with the orthologous genes being more prevalent abundant in the evolutionary process.

### Tissue-specific expression profile of *MYB5* homologous genes in *B. napus*

**Fig. 4:**
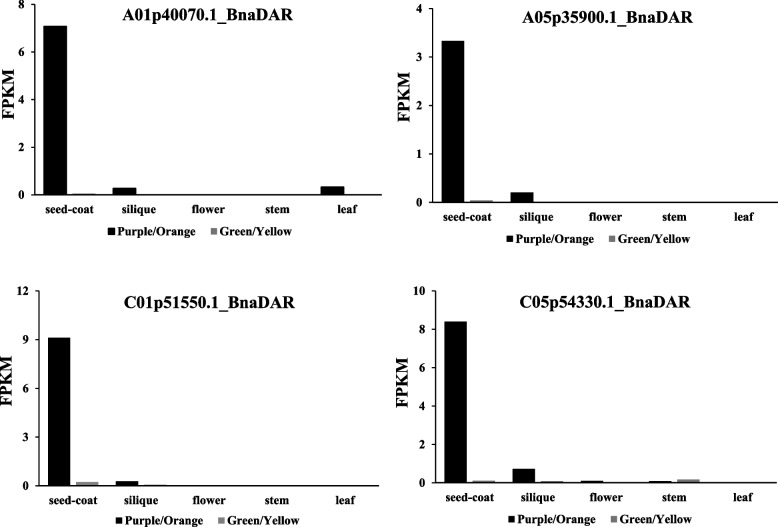
Expression analysis of four *MYB5* homologous genes in *Brassica napus* five different tissues. The FPKM (Fragments Per Kilobase Million) value obtained from transcriptome sequencing data analysis was used to represent the expression pattern of the four copies of the *MYB5* gene in different tissues, both five different tissues with three biological replicates

In order to comprehensively investigate the expression profile of *MYB5* homologous genes, we conducted an analysis on the expression patterns of four specific *MYB5* homologous genes (*A01p40070.1_BnaDAR*, *A05p35900.1_BnaDAR*, *C01p51550.1_BnaDAR*, and *C05p54330.1_BnaDAR*) in five distinct tissues of *B. napus*. Based on their expression levels (Fig.  4), the expression patterns of *MYB5* homologous genes can be broadly categorized into three groups: high expression, low expression, and minimal expression. The expression levels of the four homologous genes of *MYB5* in *B. napus* varied across different tissues. They exhibited high expression in purple seed coat, low expression in purple siliques, and minimal expression in orange-red petals and purple stems. Conversely, these four *MYB5* homologous genes showed negligible expression in yellow seed coats and petals, green leaves, stems, and siliques. Notably, *A01p40070.1_BnaDAR* displayed low expression in purple leaves, whereas the remaining MYB5 homologous copies exhibited minimal expression. The findings of this study suggest that the four *MYB5* homologous genes in *B. napus* exhibit predominant expression in the seed coat and likely play a role in regulating of PA biosynthesis in seeds. However, it is worth noting that the expression levels of these genes vary across different tissues.

### Analysis of *MYB5* homologous genes expression patterns related to PA biosynthesis in *B. napus*

**Fig. 5:**
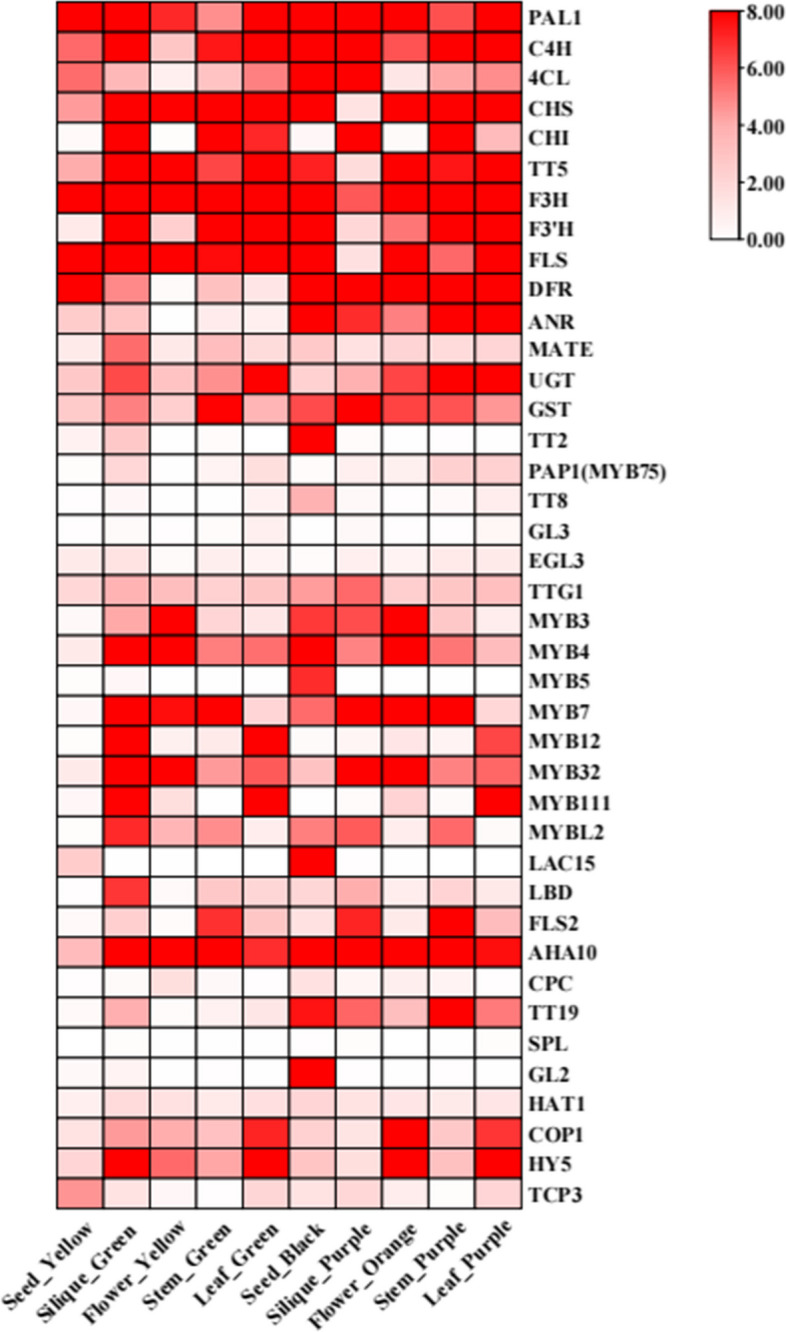
The expression patterns of anthocyanin-related genes in five tissues of *B. napus* were analyzed using a heat map that represents the FPKM values of these genes. The colors on the heat map, ranging from red to pink and white, indicate the expression levels from high to low

In order to gain a deeper understanding of the expression patterns of *MYB5* homologous genes associated with PA biosynthesis in *B. napus*, we conducted RNA-seq analysis on five different tissues: seed coat, pod, flower, stem, and leaf. Subsequently, we obtained relevant FPKM expression data. By utilizing the anthocyanin biosynthesis genes previously reported in *A. thaliana* as a reference, we identified the PA-related genes in *B. napus* through a two-way BLAST analysis. Based on the expression data of the PA-related genes across the five tissues, we employed TBtools software to generate an expression heat map (Fig. 5, Supplementary Table S6). In nearly all tissues, there was notable upregulation of genes associated with the phenylpropane synthesis pathway and flavonol synthesis pathway, including *PAL1*, *C4H*, *4CL*, *CHS*, *CHI*, *F3H*, *F3H*, and *FLS*. Additionally, genes related to the biosynthesis of PA namely *DFR*, *ANS*, *ANR*, *UGT*, and *GST*, exhibited significant upregulation specifically in purple or orange organs where PA synthesis and accumulation occurred. Particularly, *ANR* (*BAN*) is highly expressed specifically in the black seed coat (Fig. 5). Furthermore, certain PA transporters, such as *TT19,* displayed high expression levels purple or orange-red tissues, while being either lowly expressed or virtually absent in yellow or green tissues. The transcriptional regulators *MYB5*, *TT2*, *TT8*, *LAC15*, and *GL2* associated with PA biosynthesis exhibited significant up-regulation in the purple-black seed coat, whereas their expression levels were comparatively low or negligible in other tissues (Fig. 5). These findings provide additional evidence supporting the specific expression in the seed coat, where it plays a regulatory role in PA biosynthesis and accumulation.

### WGCNA analysis of genes related to PA biosynthesis in different tissues of *B. napus*

**Fig. 6:**
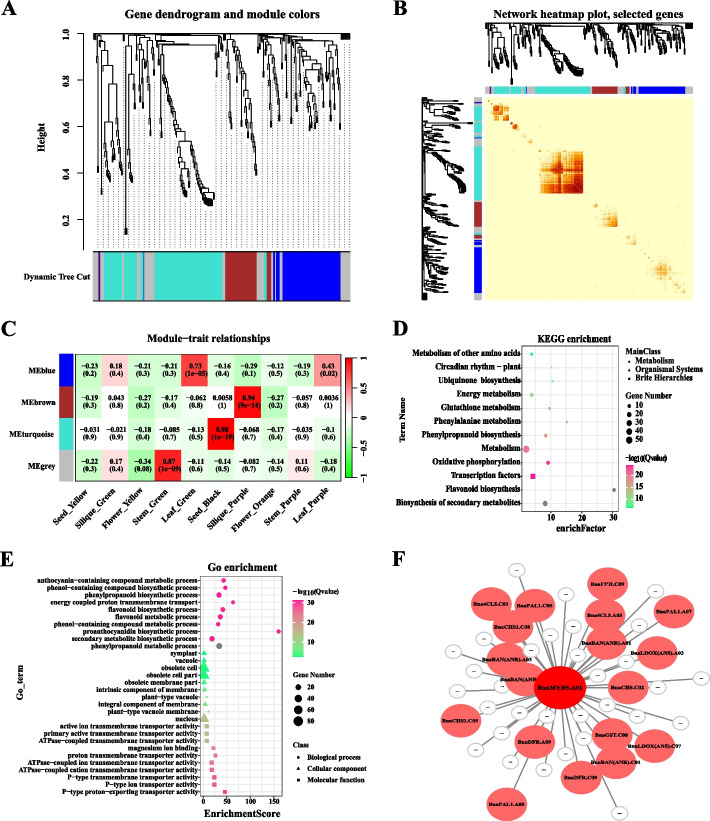
Co-expression analysis of genes related to anthocyanin biosynthesis was conducted in five tissues of *B. napus*. **A** Modular hierarchical clustering: The co-expression modules are depicted in different colors, while gray modules indicate no correlation between genes. **B** Module gene clustering heatmap: The gene expression network of anthocyanin biosynthesis-related genes in different tissues was analyzed using WGCNA, leading to the clustering of genes into distinct co-expression modules. **C** Module-to-sample correlation heatmap: Correlation analysis was performed between the co-expression modules of various genes associated with anthocyanin biosynthesis in different tissues. The numbers above the heat map indicate the Pearson correlation coefficient (r) values. **D** KEGG enrichment analysis of ‘turquoise’ module. **E** GO enrichment analysis of ‘turquoise’ module. **F** Cytoscape representation of co-expression network of the hub gene in the ‘turquoise’ module

In order to investigate the key genes associated with PA biosynthesis in various tissues of *B. napus*, we conducted a weighted gene co-expression network analysis (WGCNA) to examine the expression modules of PA biosynthesis-related genes across five tissues (Fig. 6, Supplementary Table S6). Specifically, we examined the expression levels of PA synthesis-related genes in five tissues of *B. napus* and generated a topological overlapping heat map. The results of the co-expression network analysis revealed that the genes involved in PA biosynthesis in *B. napus* were categorized into four distinct co-expression modules (Fig 6A). Following this, we conducted an analysis of the correlation heatmap between modules and samples. The results indicate that the ‘turquoise’ module exhibits the strongest connectivity (Fig. 6B). Additionally, when examining the correlation between modules and PA biosynthesis-related genes across 5 tissues, we identified that 4 sample-specific modules (module-sample correlations > 0.65 or < - 0.65, *p*-values < 0.05). Notably, the ‘blue’ module (module-trait correlations = 0.73, *p*-values = 1e-05) in green leaves the ‘brown’ module (module-trait correlations = 0.94, *p*-values = 9e-14) in purple siliques, he ‘turquoise’ module (module-trait correlations = 0.98, *p*-values = 1e-19) in black seed coats and the ‘grey’ module (module-trait correlations = 0.87, *p*-values = 1e-09) in green stems display a significant positive correlation (Fig. 6C). Significantly, the ‘turquoise’ module characterized by black seed coats exhibits the strongest correlation relationship suggesting a high degree of similarity in the expression patterns of genes involved in PA biosynthesis within the seed coat.

In addition, an analysis of GO and KEGG enrichment was conducted utilizing the 114 genes belonging to the 'turquoise' module (Supplementary Table S7). The KEGG enrichment outcomes indicate that metabolism and transcription factors exhibit the highest number of enriched genes, with the flavonoid biosynthesis entry displaying the highest enrich factor value (Fig. 6D). Similarly, the GO enrichment results reveal that the PA biosynthetic process entry attains the highest enrichment score value (Fig. 6E). These findings provide additional evidence supporting the close association between the 'turquoise' module and the metabolic regulation of PA biosynthesis. This suggests that the 'turquoise' module may exhibit the highest correlation with the development of black seed coat in rapeseed. In order to gain a deeper understanding of the relationship between genes within the module and the identification of hub genes (genes with high connectivity), a correlation network was constructed for the 'turquoise' module. In the 'turquoise' module, a total of 50 genes (copies) that are associated with the biosynthesis of proanthocyanidins (PAs) exhibited robust regulatory relationships. These genes include *PLA1*, *4CL5*, *CHS1*, *F3'H*, *DFR*, *ANS*, *ANR* and *GST*, which are structural genes involved in the PA biosynthesis pathway. Additionally, there were 32 transcriptional regulators identified that are linked to PAs biosynthesis (Fig. 6F, Supplementary Table S8). Notably, MYB5 and BAN (ANR) demonstrated the highest degree of correlation (Supplementary Table S8). These highly correlated genes are referred to as hub genes within the gene co-expression network and play a critical role in the analysis of PAs biosynthesis regulation in the seed coat of *B. napus*.

### Protein-protein interaction network analysis of ‘turquoise’ module genes

**Fig. 7:**
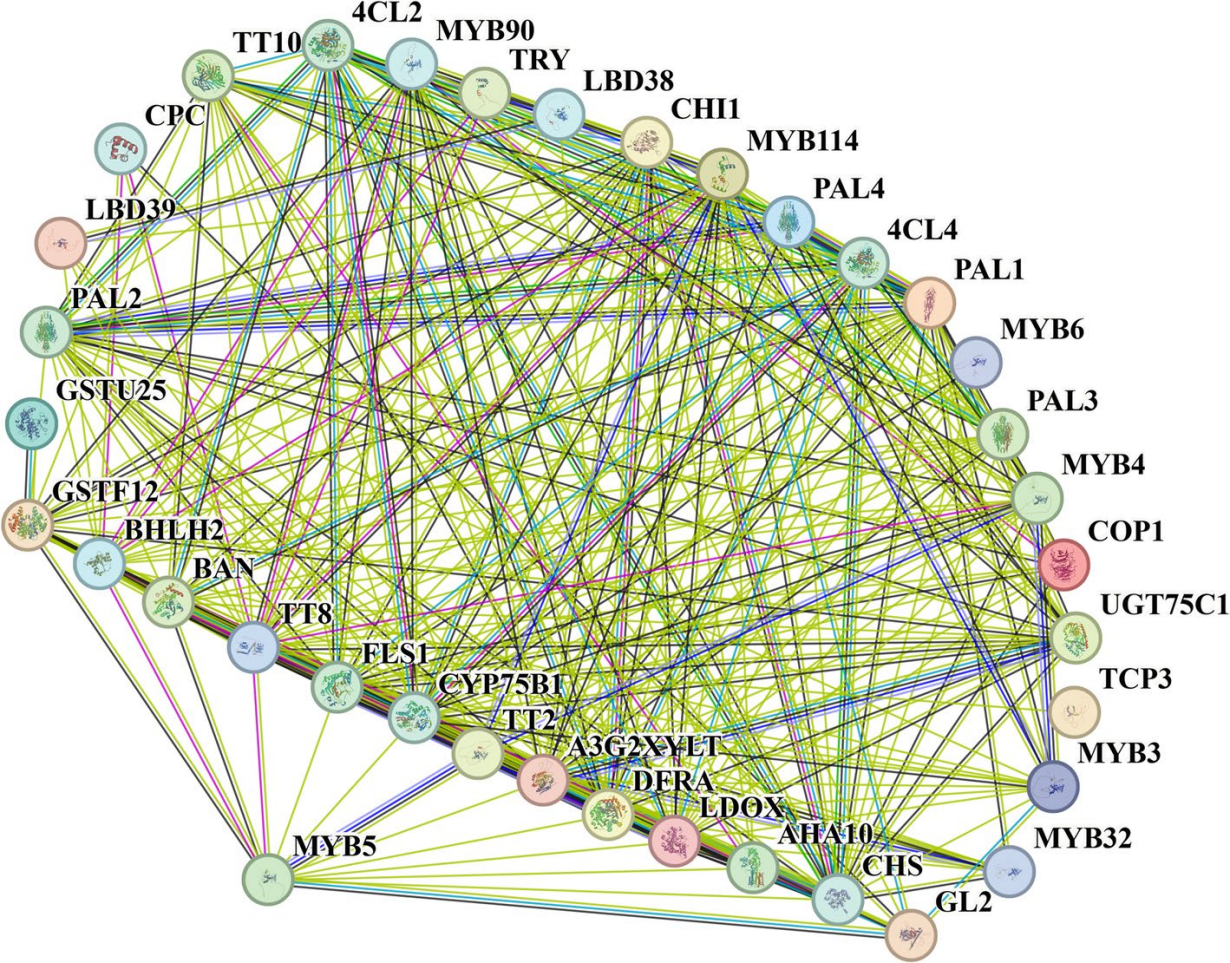
Protein-protein interaction network analysis of ‘turquoise’ module genes. Interactions between the proteins are represented by connecting lines, with the line thickness reflecting the strength of the interaction

Protein-protein interaction networks serve as valuable and practical resources for systematically investigating the assembly of protein complexes or signaling cascades. To gain deeper insights into the association between the genes in the core module and the screen hub genes, the interaction network of the ‘turquoise’ module was predicted using the *A. thaliana* homologous gene map and the online STRING website (Fig. 7). The findings revealed direct interactions and connections between MYB5 and GSTF12, BHLH2, BAN(ANR), TT8, FLS1, CYP75B1, TT2, A3G2XYLT, DFRA, LDOX(ANS), AHA10, CHS(TT4) and GL2, with distinct interaction modes observed for GSTF12, GL2, and TT8 (Fig. 7). Specifically, MYB5 exhibits a robust and direct interaction with CHS, CHI, DFR, and ANS. Notably, CHS is early catalytic proteins involved in the biosynthesis pathway of PAs, facilitating the production of flavonoids. Conversely, LDOX(DFR) and BAN(ANR) serve as the late catalytic proteins in the PA biosynthesis pathway, enabling the conversion of flavonoids into PAs. This further substantiates the pivotal role of MYB5 as a key transcription factor governing PA biosynthesis in the seed coats of *B. napus*. Furthermore, MYB5 has the ability to directly engage with the bHLH-like transcription factor TT8, exhibiting two distinct interaction modes. This suggests that MYB5 potentially collaborates with TT8 to recruit WD40 protein, thereby facilitating the formation of a prototypical MBW transcriptional regulatory complex. This intricate networkis believed to play a pivotal role in the involvement of transcriptional regulation of PAs.

## Discussion

Brassicaceae plants exhibit range of diversity and significant variation, with certain species having undergone processes such as hybridization, polyploidization, and breeding selection [48]. Consequently, these plants serve as excellent subjects for investigating alterations in the quantity of homologous genes, evolutionary patterns, and functional differentiation [48]. Currently, owing to advancements in sequencing technology and the decrease in sequencing expenses, a comprehensive compilation of reference genome data for 31 distinct species of Brassicaceae has been published [31]. These species encompass diverse evolutionary lineages and ploidy levels within the Brassicaceae family [31], thereby furnishing valuable data for our investigation into the evolutionary patterns of the *MYB5* gene. The primary objective of this study is to perform a genome-wide identification and phylogenetic analysis of the *MYB5* gene across Brassicaceae species possessing available genome information. The transcriptome data of various tissues of *B. napus* were utilized to conduct a comprehensive analysis of the tissue-specific expression of *MYB5* and its role in the regulation of PA transcription. To achieve this objective, a systematic examination of *MYB5* was performed, encompassing duplication events, genetic evolution, motif distribution, promoter cis-acting elements, tissue-specific expression, and expression patterns. The findings presented in this study reveal the identification of a total of 51 homologous genes of *MYB5* from 31 within the Brassicaceae species family, with *B. napus* prosses 4 homologous copies. The analysis of expression patterns revealed a significant association *MYB5* and genes related to PA biosynthesis in the seed coat of *B. napus*. All 4 homologous genes exhibited specific and high expression levels, suggesting that *MYB5* may serve as a crucial transcription factor in regulating PA biosynthesis in the seed coat. These findings will be further discussed.

### Evolution of *MYB5* in Brassicaceae species

Over the course of their evolutionary trajectory, Brassicaceae species have experienced diploidization, triploidization, and natural hybridization occurrences, leading to a wide array of variations, particularly within the six *Brassica* crops situated in the U's triangle [48–52]. The evolution of *MYB5* in the Brassicaceae was found to be synchronized with the overall genome evolution. This synchronization was observed in conjunction with polyploidization and hybridization events, which led to changes in both the copy number and gene function of homologous genes (Fig. 1). Our findings indicate that among the 31 species included in our study, the distribution of *MYB5* homologous genes varied. Specifically, 21 species had a single copy, 4 species had two copies, 3 species in 3 copies, 2 species had 1 copy, and 1 species had 5 copies (Table 1). These variations in copy number of *MYB5* homologous genes are closely associated with polyploidization and hybridization events. In addition, an analysis was conducted on the alterations in copy number and evolutionary relationship of *MYB5* homologous genes across six species of *Brassica*. The diploid species *B. rapa* (AA genome), *B. oleracea* (CC genome) and *B. nigra* (BB genome) exhibited copy numbers of 1, 2 and 3, respectively. Conversely, the allotetraploid species *B. juncea* (AABB genome), *B. napus* (AACC genome) and *B. carinata* (BBCC genome) displayed copy numbers of 5, 4, and 4, respectively (Table 1). The findings indicate that the allotetraploid species' genome undergoes intricate chromosomal rearrangements rather than a straightforward combination of two diploid species. Furthermore, the constructed phylogenetic tree, based on MYB5 homologous proteins, exhibits a strong alignment with the evolutionary relationship of Brassicaceae as reported by Nikolov et al. [47].

Furthermore, an analysis was conducted on the conserved domains of 51 homologous proteins of MYB5, a transcription factor belonging to the R2R3-MYB type. Subsequently, an examination was performed to assess the selection pressure on both orthologs and paralogs of *MYB5* (Fig. 3). The findings revealed that *MYB5* orthologous genes were present in 31 species of the Brassicaceae family, while paralogous genes were identified in 10 species of the same family (Supplementary Table S2). The Ks value of both orthologous and paralogous genes exhibited a significantly higher magnitude compared to the Ka value, suggesting a pronounced prevalence of synonymous substitutions in the *MYB5* homologous gene. This observation implies a relatively conservative evolutionary pattern. Furthermore, the Ka/Ks values for all 2467 gene pairs were consistently less than 1, indicating that the interspecies evolutionary pressure on *MYB5* homologous genes primarily stemmed from purifying selection. Additionally, the variation in orthologous genes appeared to be more abundant throughout the course of evolution. Furthermore, the results ofthe correlation analysis for Ka, Ks, and Ka/Ks values provided additional evidence that the correlation coefficient of orthologous genes was significantly higher than that of paralogous genes. This finding suggests that the synonymous substitution of orthologous genes played a more substantial role in the evolutionary process of *MYB5*.

### *MYB5* may specifically regulate the formation of seed coat color

In Brassicaceae species, the seed coat colors predominantly consist of yellow and black, with some intermediate shades. Yellow seeds are generally regarded as superior in quality due to their thinner shells, higher oil and protein content, and lower levels of fiber, pigments, and polyphenols [53]. Breeding yellow-seeded rapeseed is a significant objective within the realm of rapeseed breeding. Over the course of several decades, researchers have on ducted extensive investigations into the formation and regulatory mechanisms underlying the yellow seed coat [24, 28–30, 54–56]. The transition from yellow to black seed coat primarily occurs as a result of pigment deposition, particularly proPAs [57]. Consequently, a thorough analysis of the transcriptional regulation mechanism governing seed coat PAs is crucial for the successful breeding of yellow rapeseed. Xu et al. [18] conducted a comprehensive investigation on the seed coat color formation mechanism in the model plant *A. thaliana*. Within the PA synthesis pathway, only *DFR*, *ANS*(*LDOX*), *BAN*, *TT12*, *TT19* and *AHA10* were identified as direct targets of the MBW complex. Notably, the TT2-TT8-TTG1 complex emerged as a key player in seed development, while MYB5-TT8-TTG1, TT2-EGL3-TTG1 and TT2-GL3-TTG1 also exhibited tissue-specific involvement [18]. In *Brassica* crops, *TT1* [27], *TT2* [28, 58], *TT8* [21, 29, 56, 59, 60] and *TT10* [26, 61] were reported to be involved in the regulation of seed coat color formation in multiple species. The findings of this study demonstrate that certain R2R3-MYB-type transcription factors (such as *TT1*, *TT2*, *TT8*, *TT10*) facilitate the recruitment of bHLH-type transcription factors (*TT8*, *GL3*, *EGL3*) and the TTG1 protein. This collaborative assembly forms a MBW complex that specifically targets the late structural genes involved in the synthesis pathway of PAs. Consequently, this regulatory mechanism plays a crucial role in t the biosynthesis and accumulation of PAs, which ultimately determines the formation of the black seed coat.

In the Brassicaceae family, *MYB5* was initially identified as being specifically expressed in *A. thaliana* seeds and playing a role in the regulation of seed coat color [18]. In *Medicago truncatula*, the interaction between *MtMYB5* and *MtMYB14* (a homolog of *TT2*) was found to synergistically activate anthocyanin reductase, thereby regulating procyanidin biosynthesis [62]. Furthermore, *MYB5* was also implicated in the regulation of the heat stress response in *A. thaliana* [63]. Interestingly, our previous study revealed differential expression of the R2R3-MYB transcription factor *MYB90* in the leaves of six *Brassica* species. This factor potentially interacts with *TT8* to regulate the expression of *F3H*, *LDOX*, *ANS*, and *UF3GT*, thereby facilitating the bioavailability of anthocyanins biosynthesis [64]. Moreover, in the seed coats of these six *Brassica* crops, the R2R3-MYB transcription factors *MYB5* and *TT2* exhibited differential expression, suggesting that they may serve as pivotal genes in the regulation of seed coat color in *Brassica* crops [30]. In this study, it was observed that four homologous copies of *MYB5* exhibited specific expression in the seed coat of rapeseed, as determined through tissue-specific analysis (Fig. 4). Both expression pattern analysis and WGCNA revealed a close association with and strong co-expression between *MYB5* and *TT8*, as well as late structural genes *ANR* involved in anthocyanin synthesis (Figs. 6 and 7). The findings from protein interaction network analysis further supported the direct targeting of transcription factors TT8 and GL2 by MYB5, along with structural genes such as CHS, CHI, DFR, ANS (LDOX) and BAN(ANR). Consequently, *MYB5* may specifically regulate *BAN*(*ANR*) expression and thus actively participate in the biological regulation of PAs. The findings of this study suggest that within *Brassica* crops, the production and buildup of anthocyanins in various tissues may exhibit tissue-specific patterns, with *MYB5* potentially exerting a significant influence on the control of seed coat color development. These findings necessitate additional validation through subsequent experimental investigations. In conclusion, the outcomes of this research contribute novel insights into the exploration of the mechanism underlying the variation in rape seed coat color mediated by *MYB5*.

## Conclusions

In this study, a total of 51 *MYB5* homologous genes were identified from 31 species within the Brassicaceae family, and through phylogenetic analysis, they were categorized into 4 distinct subfamilies. The analysis of Ka/Ks ratios revealed that the interspecific evolution of the *MYB5* gene is primarily driven by purifying selection acting on homologous genes, with Ks exerting a greater influence on the selection pressure. By investigating the expression of the *MYB5* gene expression in various tissues of *B. napus*, including stems, leaves, petals, siliques and seed coats, we hypothesized that *MYB5* plays a specific role in regulating PAs biosynthesis in the seed coats. The findings from expression pattern analysis and WGCNA further supported the proposition that *MYB5* exhibits high and specific expression in the seed coat and is closely associated with genes involved in PA biosynthesis. At the same time, by searching for module hub genes, our findings indicate a strong correlation and regulatory relationship between *MYB5* and *BAN*(*ANR*). Additionally, the analysis of the protein-protein interaction network revealed significant interactions between MYB5 and BAN (ANR), LDOX (ANS), TT2, TTT8, and other structural genes and transcriptional regulatory proteins involved in the PA biosynthetic pathway. These results suggest that *MYB5* may be specifically expressed in the seed coat of *B. napus* to drive the expression of *BAN* (*ANR*), facilitate PA biosynthesis, and regulate the color formation of rapeseed seed coat. Nevertheless, additional experimental validation is imperative to confirm the veracity of these findings.

### Supplementary Information

**Additional file 1: Supplementary Table 1**. MYB5 homologous and their sequence in 31 Brassicaceae species. 

**Additional file 2: Supplementary Table 2.** *MYB5* homologous in 31 Brassicaceae species.

**Additional file 3: Supplementary Table 3.** *Cis*-element analysis of MYB5.

**Additional file 4: Supplementary Table 4.** Selection pressure analysis of *MYB5.*

**Additional file 5: Supplementary Table 5.** The expression patterns of anthocyanin-related genes in different tissues of *B. napus*.

**Additional file 6: Supplementary Table 6.** The FPKM values in different tissues of *B. napus.*

**Additional file 7: Supplementary Table 7.** The genes of ‘turquoise’ module.

**Additional file 8: Supplementary Table 8**. The hub gene in the ‘turquoise’ module.

## Data Availability

All RNA-seq data in this study were downloaded from the NCBI (https://www.ncbi.nlm.nih.gov/), with biological projects PRJNA554517 (leaves), PRJAN855492 (stems, flower petals), PRJNA734925 (siliques), and PRJNA597958 (seeds).

## Abbreviations

PAL: Phenylalanine ammonia-lyase
C4H: Cinnamate-4-hydroxylase
4CL: 4-coumarate CoA ligase 4
CHS: chalcone synthase
CHI: Chalcone isomerase
F3H: Flavanone 3-hydroxylase
FLS: Flavonol synthase
DFR: Dihydroflavonol 4-reductase
ANS: Anthocyanidin synthase
BAN: Anthocyanidin oxidoreductase
AT: Acyltransferase
GT: Glucosyltransferase
UFGT: UDP-flavonoid glucosyltransferase
TT2: TRANSPARENT TESTA 2
